# Longitudinal Changes in Maternal Depressive and Anxiety Symptoms Following COVID-19 During Pregnancy: A Cohort Study from Slovakia

**DOI:** 10.3390/jcm15103931

**Published:** 2026-05-20

**Authors:** Cecilia Holikova, Adriana Goldbergerova, Miroslav Borovsky, Lubomira Izakova, Jan Mikas, Jana Namesna, Zuzana Kristufkova, Michal Illovsky, Alexandra Kristufkova

**Affiliations:** 1 First Department of Obstetrics and Gynaecology, Faculty of Medicine, St. Cyril and Method’s Hospital, Comenius University Bratislava and University Hospital Bratislava, Antolská 11, 851 07 Bratislava, Slovakia; marcisova15@uniba.sk (C.H.); kolekova7@uniba.sk (A.G.); borovsky@pe.unb.sk (M.B.); alexandra.kristufkova@fmed.uniba.sk (A.K.); 2 Department of Psychiatry, Faculty of Medicine, Old Town Hospital, Comenius University Bratislava and University Hospital Bratislava, Mickiewiczova 13, 813 69 Bratislava, Slovakia; 3 Public Health Authority of the Slovak Republic, Trnavská Cesta 52, 826 45 Bratislava, Slovakia; jan.mikas@gmail.com; 4 Regional Public Health Authority of the Slovak Republic, Cesta k Nemocnici 1, 975 56 Banská Bystrica, Slovakia; jana.namesna@uvzsr.sk; 5 Institute of Epidemiology and Prevention, Faculty of Public Health, Slovak Medical University, Limbová 12, 833 03 Bratislava, Slovakia; zuzana.kristufkova@szu.sk; 6 Independent Researcher, Bratislava, Slovakia; michal.illovsky@gmail.com

**Keywords:** COVID-19, pregnancy, postpartum, Edinburgh Postnatal Depression Scale, perinatal mental health

## Abstract

**Background/Objectives**: The COVID-19 pandemic has raised concerns about maternal mental health, particularly among women infected during pregnancy. This study aimed to examine longitudinal changes in depressive and anxiety symptoms and subjective distress among pregnant women in Slovakia with confirmed SARS-CoV-2 infection and to explore the role of obstetric complications and vaccination status in these trajectories. **Methods**: In this retrospective longitudinal study, women with SARS-CoV-2 infection during pregnancy were assessed at three time points: during infection, six weeks postpartum, and one year postpartum (11 March 2020–5 May 2023). Depressive symptoms were measured using the Edinburgh Postnatal Depression Scale (EPDS; cut-off ≥ 11), anxiety symptoms were measured using the EPDS-3A subscale (cut-off ≥ 5), and subjective distress was measured using visual analogue scales (VAS). A repeated-measures ANCOVA design was used to evaluate within-subject changes over time while adjusting for vaccination status and pregnancy complications. **Results**: Of 1184 contacted women, 170 provided complete data. The proportion of women exceeding the EPDS cut-off decreased from 27.6% during infection to 17.6% at six weeks postpartum and 4.7% at one year postpartum. Anxiety symptoms showed a similar pattern, declining from 27.6% during infection to 20.6% at six weeks postpartum and 7.6% at one year postpartum. Repeated-measures analyses confirmed significant time effects across psychological outcomes, with symptom levels decreasing over the postpartum year. Post-infection obstetric complications were associated with higher subjective distress at selected time points. **Conclusions**: Psychological symptoms were highest during the acute infection period and declined significantly over time. These findings support the importance of timely mental health screening during pregnancy affected by COVID-19, while suggesting that, in many women, psychological distress may decrease across the postpartum year.

## 1. Introduction

The COVID-19 pandemic has had an unprecedented impact on global health care systems and has profoundly affected mental health worldwide [1,2]. Pregnant women may be particularly vulnerable to psychological distress due to the complex interplay between the physical, hormonal, and psychological changes associated with pregnancy and the additional stressors imposed by the pandemic [3]. In Slovakia, as in other countries, pregnant women faced increased uncertainty, disrupted health services, and increased social isolation, all of which are known contributors to increased psychological distress during pregnancy [4].

Prevalence of unipolar depression across pregnancy trimesters is reported at 7.4% in the first trimester, 12.8% in the second trimester, and 12% in the third trimester [3]. Depression in the perinatal period, including the postpartum form, is assessed using the Edinburgh Postnatal Depression Scale (EPDS) [5].

The prevalence of generalized anxiety disorder in the perinatal period is estimated at 8.5–10.5%, while in the postpartum period its prevalence ranges from 4.4 to 10.8% [6]. A standardized screening method includes the GAD-7 (Generalized Anxiety Disorder-7), but the equally validated EPDS-3A (subscale of the EPDS) may also be used [7].

Both conditions may have negative impacts, including low birth weight, increased risk of preterm birth, and long-term complications affecting the child’s future neurobiological development [8,9,10,11].

The COVID-19 pandemic represented an external stressor exerting a substantial negative impact on mental health. A systematic review and meta-analysis including 46 studies and nearly 48,000 participants found a prevalence of clinically significant prenatal depressive symptoms of 25.6% and anxiety symptoms of 30.5% during the COVID-19 pandemic [12]. Moreover, a meta-analysis by Ghazanfarpour et al. [13] contributed further evidence highlighting intercontinental variations in the prevalence of depression and anxiety. Their continent-based analysis showed that the prevalence of anxiety was higher in Western countries (38%) than in Asian countries (7.8%).

Furthermore, monitoring longitudinal changes in mental well-being is key to better understanding the course of mental health issues, identifying periods of risk, and effectively timing therapeutic interventions. This approach allows for better identification of high-risk periods and more targeted mental health support. One of the first longitudinal studies published was conducted by López-Morales et al. [14], who monitored 102 pregnant and 102 non-pregnant women at three points in time during the first wave of the pandemic. Their results showed that pregnant women experienced higher levels of depression and anxiety across the three monitored periods compared to non-pregnant women. However, longitudinal data specifically examining the trajectory of psychological symptoms following confirmed SARS-CoV-2 infection during pregnancy remain limited, particularly in Central and Eastern European populations.

The aim of this study was to examine longitudinal changes in depressive and anxiety symptoms and subjective distress in women following SARS-CoV-2 infection during pregnancy.

We hypothesized that (1) depressive and anxiety symptoms would be highest during acute infection and decrease over time, and (2) pregnancy-related complications would be associated with higher levels of depressive and anxiety symptoms.

## 2. Materials and Methods

This study is a retrospective cohort study with repeated measurements. The study included pregnant women living in Slovakia during the COVID-19 pandemic who had overcome SARS-CoV-2 infection during pregnancy. The study examined changes in depressive symptoms, anxiety symptoms, and subjective distress at three time points: during SARS-CoV-2 infection, six weeks postpartum, and one year postpartum. The study was conducted within the national COVID-19 PreMatOut (COVID-19 Pregnancy & Maternal Outcome) project. Eligible participants were identified through national public health records provided by the Public Health Authority of the Slovak Republic. Women were contacted by telephone and, upon providing written informed consent, received the self-administered study questionnaire electronically via e-mail or by post. All procedures were conducted in accordance with the ethical standards of the institutional research committee and with the 1964 Helsinki Declaration and its later amendments. The study was approved by the Ethics Committee of the Faculty of Medicine, Comenius University and University Hospital in Bratislava (May 2021). A total of 1184 pregnant women who tested positive for SARS-CoV-2 were retrospectively identified and contacted for participation in the study. A formal a priori sample size calculation was not performed, as the study was exploratory in nature and based on all available eligible participants with complete repeated measurements across all three assessment periods. The study was reported with consideration of the STROBE reporting recommendations for observational studies.

The inclusion criteria were as follows: confirmed SARS-CoV-2 infection during pregnancy, availability of a valid telephone contact, and provision of informed consent. Only participants who provided complete data across all three assessment periods were included in the longitudinal analyses (complete-case approach).

Women who failed to meet the inclusion criteria or to provide complete data necessary for analysis were excluded from the study.

Data collection was initiated after ethical approval in 2021 and continued until May 2023. The study retrospectively covered the pandemic period from March 2020 to May 2023. Participants entered the study at different time points during the pandemic, while psychological outcomes were retrospectively assessed across the three predefined study periods. The number of COVID-19 symptoms during the acute phase was recorded and categorized ordinally (0; 1–2; 3–4; 5–6; ≥7). Hospitalization was analysed separately as a binary indicator of severe disease.

Mental health was assessed using the EPDS, visual analogue scales (VAS), and open questions at three time points: at the time of the initial infection, six weeks postpartum, and one year after delivery. A validated Slovak-language version of the Edinburgh Postnatal Depression Scale (EPDS), demonstrating good psychometric properties in postpartum Slovak women, was used [15].

Depressive symptoms were assessed using the EPDS. A cut-off ≥ 11 was used to indicate clinically relevant depressive symptoms, based on meta-analytic evidence optimizing sensitivity and specificity [5].

Anxiety symptoms were evaluated using the EPDS-3A subscale (calculated as the sum of items 3, 4, and 5 of the EPDS, with a range of 0–9 points). A cut-off ≥ 5 was used to indicate clinically relevant anxiety symptoms, based on validation data demonstrating optimal diagnostic accuracy [7].

Three VAS measures were used to assess subjective well-being (VAS 1), fear (VAS 2), and perceived maternal inadequacy (VAS 3). Scores ranged from 0 to 10, with higher values indicating greater symptom intensity. VAS scores were analysed as continuous variables.

Statistical analyses were performed using IBM SPSS Statistics version 23.0 (IBM Corp., Armonk, NY, USA). To evaluate changes over time while adjusting for covariates, a repeated-measures ANCOVA was conducted. This approach was selected to evaluate within-subject changes across the three assessment periods (infection, six weeks postpartum, one year postpartum) while adjusting for clinically relevant covariates in participants with complete repeated measurements. The dependent variables were the total EPDS score and the VAS scores. SARS-CoV-2 infection severity was included as a between-subject factor, operationalized according to the number of reported symptoms, with hospitalization analysed as a separate category. Covariates included COVID-19 vaccination status, pregnancy complications prior to infection, pregnancy complications following infection, and delivery complications. The assumption of sphericity was assessed using Mauchly’s test; when violated, Greenhouse–Geisser corrections were applied. Homogeneity of variances for between-subject factors was assessed using Levene’s test.

The main effects of time and disease severity, as well as interactions between time and disease severity, were evaluated. Effect sizes were expressed as partial eta squared (η*p*^2^), with values of 0.01, 0.06, and 0.14 interpreted as small, medium, and large, respectively. Statistical significance was set at *p* ≤ 0.05. The total score of the EPDS was included in the repeated-measures design. The EPDS-3A anxiety subscale was analysed separately using the same repeated-measures design specification.

## 3. Results

Between March 2020 and May 2023, the Public Health Authority of the Slovak Republic identified 1184 women with confirmed SARS-CoV-2 infection during pregnancy. Of these, 901 women agreed to participate following telephone contact, and 170 provided complete data and were included in the final longitudinal analyses. The mean maternal age was 36.1 ± 4.9 years. Most infections occurred in 2021 (58.2%), and 42.9% of women had received at least one dose of a COVID-19 vaccine prior to infection (regardless of completion of the full vaccination schedule). Detailed characteristics of the study sample are presented in Table 1.

Repeated-measures ANCOVA revealed a significant effect of time across all psychological outcomes (Table 2). Results are reported with Greenhouse–Geisser correction where the assumption of sphericity was violated.

**Figure 1:**
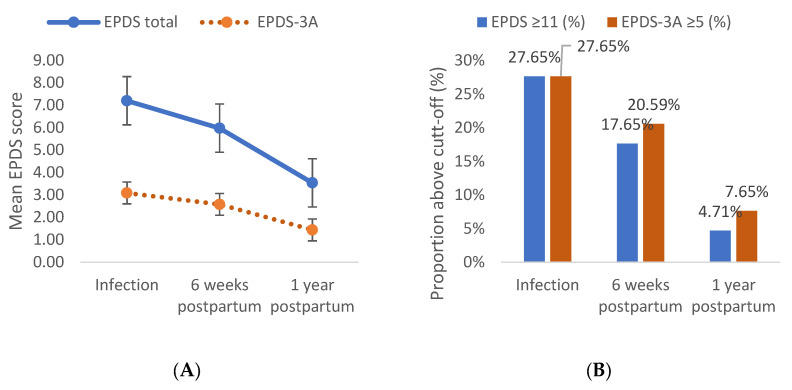
Longitudinal trajectories and clinical thresholds: prevalence of depression and anxiety symptoms among women infected with SARS-CoV-2 during pregnancy. (**A**) EPDS scores over time. (**B**) Participants above clinical cut-off.

Depressive symptoms (EPDS) decreased significantly over time (F(1.671, 282.400) = 9.13, *p* < 0.001, η*p*^2^ = 0.054), representing a small-to-moderate effect size. Anxiety symptoms assessed using the EPDS-3A subscale also showed a significant effect of time (F(1.794, 303.136) = 5.83, *p* = 0.005, η*p*^2^ = 0.035), as illustrated in Figure 1A. Similar time effects were observed for subjective well-being (VAS 1: F(2.000, 338.000) = 23.13, *p* < 0.001, η*p*^2^ = 0.126), fear (VAS 2: F(1.912, 323.159) = 38.21, *p* < 0.001, η*p*^2^ = 0.193), and perceived maternal inadequacy (VAS 3: F(1.849, 312.406) = 5.98, *p* = 0.004, η*p*^2^ = 0.036).

In addition, the proportion of women exceeding the clinical cut-off for depressive symptoms (EPDS ≥ 11) decreased from 27.65% during infection to 17.65% at six weeks postpartum and 4.71% at one year postpartum (Figure 1B). A similar pattern was observed for anxiety symptoms assessed using the EPDS-3A subscale (≥5), with prevalence declining from 27.65% during infection, to 20.59% at six weeks postpartum, and to 7.65% at one year postpartum. These findings indicate a marked reduction in the proportion of women at risk for clinically relevant depressive and anxiety symptoms over the first postpartum year. Considerable overlap between clinically relevant depressive symptoms (EPDS ≥ 11) and anxiety symptoms (EPDS-3A ≥ 5) was observed across all assessment periods. The proportion of participants meeting criteria for both depressive and anxiety symptoms was 21.18% during SARS-CoV-2 infection, 16.47% at six weeks postpartum, and 4.12% at one year postpartum.

### 3.1. Effect of Obstetric Complications and Vaccination

The repeated-measures model included time as a within-subject factor, with vaccination status and obstetric complications (before infection, after infection, and during delivery) included as covariates.

No significant associations were observed between vaccination status and EPDS or EPDS-3A scores at any time point (all *p* > 0.05). Similarly, obstetric complications occurring before or after infection, as well as complications during delivery, were not significantly associated with depressive or anxiety symptom severity across the three assessments (all *p* > 0.05).

For subjective well-being (VAS 1), vaccination status was associated with lower distress at six weeks postpartum (B = −0.909, *p* = 0.041, η*p*^2^ = 0.026). In contrast, obstetric complications occurring after infection were associated with higher distress at six weeks postpartum (B = 1.550, *p* = 0.004, η*p*^2^ = 0.052). No other significant effects were observed.

Regarding fear-related symptoms (VAS 2), vaccination was associated with lower fear scores at six weeks postpartum (B = −0.906, *p* = 0.029, η*p*^2^ = 0.030), whereas slightly higher scores were observed at one year postpartum (B = 0.599, *p* = 0.044, η*p*^2^ = 0.025), although effect sizes remained small. Obstetric complications after infection were associated with increased fear during the infection period (B = 1.335, *p* = 0.013, η*p*^2^ = 0.038) and at six weeks postpartum (B = 1.129, *p* = 0.022, η*p*^2^ = 0.032).

For perceived maternal inadequacy (VAS 3), vaccination was associated with lower scores at six weeks postpartum (B = −0.888, *p* = 0.024, η*p*^2^ = 0.032). Obstetric complications after infection were associated with lower perceived inadequacy at one year postpartum (B = −0.642, *p* = 0.038, η*p*^2^ = 0.027), although effect sizes were small.

### 3.2. Impact of SARS-CoV-2 Infection Severity (Number of Symptoms)

No significant between-subject effect of SARS-CoV-2 infection severity was observed for any psychological outcome (see Table S1 in the Supplementary Materials). Neither linear nor quadratic polynomial contrasts reached statistical significance, indicating the absence of a dose–response association.

## 4. Discussion

The present retrospective longitudinal study indicates that SARS-CoV-2 infection during pregnancy was associated with increased psychological distress during the acute phase; however, symptoms declined substantially over the first postpartum year. The proportion of women exceeding clinical screening thresholds decreased over time, suggesting that the observed distress may reflect a predominantly time-limited stress response rather than persistent psychological distress in most participants. Notably, infection severity, as defined by the number of symptoms, was not associated with long-term psychological outcomes. Vaccination status and obstetric complications demonstrated only limited and time-specific associations, indicating that these factors did not fundamentally determine the overall longitudinal pattern of maternal mental health.

The elevated distress observed during the acute infection period aligns with findings from large-scale global modelling studies demonstrating a substantial increase in depressive and anxiety disorders during the COVID-19 pandemic, particularly among women [1]. Meta-analytic evidence further indicates that pregnant women experienced markedly increased rates of clinically significant depressive and anxiety symptoms during this period [2,12,16,17,18]. However, much of the available literature relies on cross-sectional assessments of pandemic-related distress. Available longitudinal studies similarly reported elevated psychological distress during the acute phases of the pandemic, with gradual symptom improvement over time among pregnant and postpartum women [14,19,20]. By focusing specifically on women with confirmed SARS-CoV-2 infection during pregnancy and employing repeated assessments across three timepoints, the present study contributes longitudinal evidence regarding the trajectory of maternal psychological symptoms beyond the acute phase. Furthermore, these findings provide data from a Central European population, a region that remains relatively underrepresented in perinatal mental health research during the pandemic.

The heightened symptom levels observed during the acute infection period may be interpreted within the broader context of uncertainty and perceived threat that characterized the pandemic environment. In pregnant women, concerns regarding potential adverse effects on foetal development, maternal health, and delivery outcomes likely intensified psychological distress [21]. Emerging longitudinal evidence suggests that uncertainty and anticipatory anxiety related to infection played a significant role in shaping psychological responses during the pandemic [22]. In this context, the elevated distress observed during infection in our cohort may reflect a stress response to perceived health threat rather than the direct somatic severity of the illness. The subsequent decline in symptoms over time may indicate psychological adaptation and stabilization during the postpartum period as uncertainty diminished [23].

From a clinical perspective, the findings suggest potential value in targeted mental health screening during acute SARS-CoV-2 infection in pregnancy [24]. Notably, 27.7% of women exceeded the clinical screening threshold for depressive symptoms (EPDS ≥ 11), and an identical proportion met the cut-off for anxiety symptoms (EPDS-3A ≥ 5) during the infection period. These figures indicate that a substantial subgroup of pregnant women may experience clinically relevant psychological distress when confronted with infection-related uncertainty. Although symptom levels declined over time, this reduction does not necessarily eliminate the need for timely psychological support during the acute phase. Early identification of women at increased risk may therefore represent a pragmatic and proportionate response to short-term psychological burden in this context. However, the longitudinal pattern of symptom decline appeared largely independent of infection severity and most examined covariates, suggesting that the observed trajectory was not primarily determined by medical or obstetric factors.

The longitudinal trajectory of psychological symptoms appeared largely independent of infection severity and most examined covariates. The number of COVID-19 symptoms and hospitalization status were not associated with depressive, anxiety, or subjective distress outcomes, and no dose–response pattern was observed. Similarly, vaccination status and obstetric complications demonstrated only limited and time-specific associations, primarily within selected subjective distress domains, with small effect sizes. These findings suggest that the observed pattern of initial distress followed by gradual decline was not primarily determined by medical severity or obstetric factors but may instead reflect a broader psychological response to the contextual stress of infection during pregnancy.

The study possesses several methodological strengths. The longitudinal design with repeated assessments across three timepoints enhances temporal interpretability of symptom trajectories. The inclusion of women with confirmed SARS-CoV-2 infection increases clinical specificity, distinguishing infection-related distress from general pandemic-related stress. Furthermore, the combined analysis of continuous symptom scores and clinically meaningful cut-off thresholds allows for both dimensional and categorical interpretation of psychological burden. The use of validated screening instruments further strengthens the reliability of the psychological measures. Finally, the study contributes data from a Central European population, a region that remains comparatively underrepresented in perinatal mental health research.

Several limitations should be considered when interpreting these findings. First, the retrospective assessment of psychological symptoms during the infection and postpartum periods may be subject to recall bias. The extended recall interval between the initial emotional experiences across assessment periods and the follow-up data collection may have influenced the accuracy of participants’ reports. Furthermore, psychological responses may also have been influenced by changing pandemic conditions across the study period. Second, obstetric complications were based on self-report and were not independently verified through medical records, which may have introduced reporting inaccuracies. Third, the use of complete-case analysis and the relatively high attrition rate may have introduced selection bias, potentially limiting the generalizability of the findings to the broader population of pregnant women affected by SARS-CoV-2 infection. In addition, incomplete demographic and clinical data from participants lost to follow-up limited the possibility of conducting a detailed attrition bias analysis. In addition, the relatively small number of hospitalized cases restricted the ability to examine the impact of severe COVID-19 courses in greater detail. The assumption of homogeneity of variances was only partially satisfied for the between-subject factor (COVID-19 severity), which may have affected the robustness of between-subject comparisons and should be considered when interpreting severity-related findings. Additionally, several potentially relevant confounding factors, including socioeconomic status, pre-existing mental health conditions, and levels of social support, were not systematically assessed and may have influenced psychological outcomes. Finally, although the longitudinal design strengthens temporal interpretation, the observational nature of the study does not permit causal conclusions regarding the relationship between infection and psychological outcomes.

In summary, the present findings indicate that psychological distress during acute SARS-CoV-2 infection in pregnancy may reflect a time-limited response to perceived health threat rather than sustained psychopathology. Although a substantial proportion of women reported clinically relevant depressive and anxiety symptoms during the infection period, symptom levels declined across the postpartum year and appeared to be largely independent of infection severity and obstetric factors. Continued research is warranted to further delineate which subgroups may remain vulnerable beyond the acute phase and to examine potential longer-term psychological outcomes following infection during pregnancy. Future studies may benefit from the use of mixed-effects models, which may provide greater flexibility in handling incomplete longitudinal data. At the same time, these findings support the value of timely mental health screening and proportionate psychological support during acute infection, while recognizing that for many women, psychological recovery appears to occur naturally over time.

## Figures and Tables

**Table 1:** Characteristics of the study sample (*N* = 170).

Characteristic	*n* (%) or Mean ± SD
**Year of infection**	
2020	43 (25.3)
2021	99 (58.2)
2022	28 (16.5)
Maternal age (years)	36.1 ± 4.9
**Parity**	
Primiparous	85 (50.0)
Multiparous	85 (50.0)
**Pregnancy complications**	
Before infection	39 (22.9)
After infection	42 (24.7)
Preterm birth	19 (11.2)
**Mode of delivery**	
Vaginally	114 (67.1)
Caesarean section	56 (32.9)
**Number of symptoms**	
Asymptomatic	8 (4.7)
1–2	16 (9.9)
3–4	49 (30.3)
5–6	45 (27.8)
7 and more	52 (32.1)
Hospitalization due to COVID-19	9 (5.6)
**Vaccination status**	
Unvaccinated	97 (57.1)
Vaccinated before infection	73 (42.9)

**Table 2:** Longitudinal changes in depressive symptoms, anxiety symptoms, and subjective distress.

Measure	Infection Mean ± SD	Six Weeks Postpartum Mean ± SD	One Year Postpartum Mean ± SD	F	*p*	Partial η*p*^2^
EPDS	7.20 ± 5.77	5.98 ± 6.59	3.54 ± 3.55	9.13	<0.001	0.054
EPDS-3A	3.09 ± 2.36	2.58 ± 2.49	1.44 ± 1.66	5.83	0.005	0.035
VAS 1	4.62 ± 3.16	2.93 ± 2.93	1.59 ± 2.18	23.13	<0.001	0.126
VAS 2	4.97 ± 2.95	2.44 ± 2.68	1.43 ± 1.85	38.21	<0.001	0.193
VAS 3	2.60 ± 2.42	2.44 ± 2.49	1.56 ± 1.68	5.98	0.004	0.036

EPDS = Edinburgh Postnatal Depression Scale; EPDS-3A = anxiety subscale of EPDS; VAS 1 = subjective well-being; VAS 2 = fear; VAS 3 = perceived maternal inadequacy.

## Data Availability

The datasets generated and analysed during the current study are available from the corresponding author on reasonable request. The data are not publicly available due to privacy and ethical restrictions.

## Supplementary Materials

The following supporting information can be downloaded at: https://www.mdpi.com/article/10.3390/jcm15103931/s1, Table S1: Between-subject effects of SARS-CoV-2 infection severity on psychological outcomes.

## Abbreviations

The following abbreviations are used in this manuscript:

COVID-19	Coronavirus disease 2019
COVID-19 PreMatOut	COVID-19 Pregnancy & Maternal Outcome study
EPDS	Edinburgh Postnatal Depression Scale
EPDS-3A	Edinburgh Postnatal Depression Scale subscale
GAD-7	Generalized Anxiety Disorder-7
SARS-CoV-2	Severe Acute Respiratory Syndrome Coronavirus 2
VAS	Visualized analogue scale
