# Supplementary material for: Longitudinal Changes in Maternal Depressive and Anxiety Symptoms Following COVID-19 During Pregnancy: A Cohort Study from Slovakia
Source: J Clin Med. 2026 May 20;15(10):3931. doi: 10.3390/jcm15103931 (PMC13208021; doi:10.3390/jcm15103931)
Supplement: Supplementary file 1 [file jcm-15-03931-s001.zip › jcm-4301405-supplementary.pdf]

Table S1. Between-subjects effects of SARS-CoV-2 infection severity on psychological outcomes

**Outcome**	**F (5,164)**	**p**	**Partial ηp^2**
EPDS	1.80	0.375	0.033
EPDS-3A	1.04	0.399	0.031
VAS 1	1.94	0.091	0.057
VAS 2	0.99	0.426	0.030
VAS 3	1.11	0.358	0.034

EPDS = Edinburgh Postnatal Depression Scale; EPDS-3A = anxiety subscale of EPDS; VAS 1 = subjective well-being; VAS 2 = fear; VAS 3 = perceived maternal inadequacy. Severity categorized by number of symptoms and hospitalization. Polynomial contrasts (linear and quadratic) were non-significant (all $p > 0.05$).
